# Estrogen replacement therapy-induced neuroprotection against brain ischemia-reperfusion injury involves the activation of astrocytes via estrogen receptor β

**DOI:** 10.1038/srep21467

**Published:** 2016-02-19

**Authors:** Yulong Ma, Hang Guo, Lixia Zhang, Liang Tao, Anqi Yin, Zhaoyu Liu, Yan Li, Hailong Dong, Lize Xiong, Wugang Hou

**Affiliations:** 1 Department of Anesthesiology, Xijing Hospital, The Fourth Military Medical University, Xi’an 710032, China; 2 Department of Anesthesiology, General Hospital of Chinese PLA Beijing Command, Beijing 100700, China; 3 First Affiliated Hospital to Chinese PLA General Hospital, Beijing 100048, China

## Abstract

The incidence of ischemic stroke is significantly increased in postmenopausal women. However, the neuroprotective effects of estrogen replacement therapy (ERT) against stroke remain controversial, and the role of astrocytes in ERT has rarely been explored. In this study, we investigated the effects of estrogen and selective estrogen receptor (ER) agonists on astrocytes activation and neuronal apoptosis in mice under conditions of cell culture oxygen and glucose deprivation and reperfusion (OGD-R), and global cerebral ischemia (GCI). We demonstrated that hippocampal astrocytes primarily express ERβ. In astrocytes, 2.5–20 nM 17β-estradiol (E2) or 10 nM DPN (ERβ agonist) not 10 nM PPT (ERα agonist), significantly increased GFAP expression. And 10 nM E2, DPN or E2+MPP (ERα antagonist), but not PPT or E2+PHTPP (ERβ antagonist), significantly reduced neuronal apoptosis following the subjection of astrocyte and neuronal cocultures to OGD-R. We also found that either 50 μg/kg E2 or 8 mg/kg DPN replacement (3 weeks) significantly increased GFAP expression and reduced GCI-induced neuronal apoptosis in hippocampal CA1 region of ovariectomized mice. These results indicate that estrogen-induced neuroprotection against ischemia-reperfusion injury involves activation of astrocytes via ERβ. Thus, the discovery and design of astrocyte-selective ERβ modulators may offer a new strategy for ERT of ischemic stroke.

The neuroprotective role of estrogen replacement therapy (ERT) remains controversial. Epidemiological studies have indicated a well-documented increase in the incidence of ischemic stroke in postmenopausal women, which is much higher than that in comparable aged men^1^. Following menopause, women suffer from a dramatic age-related decrease in serum estrogen levels, which are approximately 1% of those in premenopausal women. Several animal studies, including ours, have verified that ERT exhibits significant neuroprotective effects against ischemic stroke in either ovariectomized (OVX) rats or OVX mice^2^,^3^,^4^. Therefore, for postmenopausal women, ERT is expected to be an effective strategy for the prevention of ischemic stroke. However, several clinical trials on ERT have found that ERT did not reduce the incidence of ischemic stroke in postmenopausal women but increased the risk of coronary heart diseases and breast cancers^5^,^6^. Many previous studies have focused on the molecular mechanisms of neuronal cell death during stroke, but this approach has uniformly failed to reduce stroke-induced damage or to improve functional recovery. Recent years, glias, including astrocytes and microglial cells have gradually become the target for estrogen treatment^7^.

Astrocytes are the most abundant non-neuronal cell type in the brain and are dynamically involved in the trophic support of neurons, metabolic and ionic homeostasis, the establishment and maintenance of the blood-brain barrier, synaptic transmission, inflammatory responses and antioxidant defense^8^. Therefore, astrocytes are considered important regulators of the survival and death of neurons and have been recognized as a new target for the prevention of ischemic stroke^9^.

Estrogen exerts its function by binding to estrogen receptor (ER) α and β (ERα and ERβ) and newly identified GPR30 receptor^10^. Previous studies have found that both ERα and ERβ are expressed in primary cultured and brain tissue astrocytes^11^,^12^. Therefore, astrocytes have the greatest potential to mediate the neuroprotective effects of estrogen. However, the definitive role of astrocytes in ERT remains unclear. Several studies have demonstrated that ERα plays an indispensable role in estrogen neuroprotection^13^, although the specific cell type was not determined. Using astrocyte- and neuron-selective ERα knockout (KO) mice, a previous study found that acute estrogen treatment exhibited neuroprotective effects against experimental autoimmune encephalomyelitis (EAE) via the action of ERα on astrocytes but not on neurons^14^, validating the critical role of astrocytes in estrogen neuroprotection. However, the role of astrocyte ERβ in ERT-induced neuroprotection is poorly understood.

A recent study found that ischemic preconditioning-induced brain ischemic tolerance involved the activation of astrocytes^15^. However, whether ERT-induced neuroprotection involves the activation of astrocytes has not been thoroughly examined. The present study aims to explore the role of astrocytes in long-term ERT-induced neuroprotection against ischemic stroke and the underlying molecular mechanism, which may provide a new strategy for ERT for ischemic stroke.

## Results

### Astrocytes of the hippocampus primarily expressed ERβ

**Figure 1:**
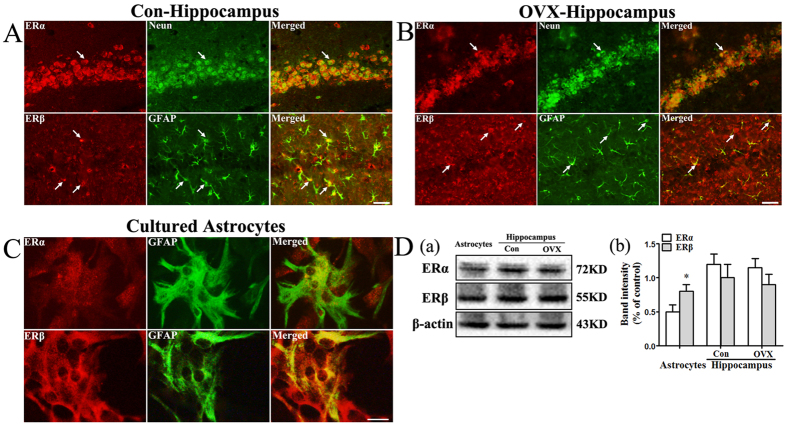
Expression of ERα and ERβ in pure cultured astrocytes and the female mice hippocampus (*n* = 5). (**A**) The location of ERα and ERβ in hippocampus of normal mice. Bar: 20 μm. (**B**) The location of ERα and ERβ in hippocampus of OVX mice. Bar: 20 μm. (**C**) The location of ERα and ERβ in cultured astrocytes. Bar: 10 μm. (**D**) (**a**) Cropped gels and blots of ERα and ERβ expression in cultured astrocytes and hippocampus detected by western blot. The samples derive from the same experiment and that gels/blots were processed in parallel. Full-length blots/gels are presented in Supplementary Fig. 4. **(b)** Data are shown as the mean ± S.D; *p < 0.05 vs. ERα expression in cultured astrocytes group.

In the hippocampus of adult normal female mice (Fig. 1A) and OVX mice (Fig. 1B), we found that ERα primarily colocalized with the neuronal marker Neun, whereas ERβ primarily colocalized with the astrocyte marker glial fibrillary acidic protein (GFAP). In primary cultured astrocytes derived from the hippocampus of newborn mice, immunofluorescence assays (Fig. 1C) indicated that both ERα and ERβ colocalized with the astrocyte marker GFAP, and relatively higher expression levels of ERβ were observed. Western blot analysis (Fig. 1D) indicated that ERβ was expressed at significant higher levels than ERα in cultured astrocytes (*p < 0.05), whereas both ERα and ERβ were highly expressed in the hippocampus of adult female mice and OVX mice.

### E2 and DPN treatment upregulated GFAP expression in primary cultured astrocytes and in the hippocampus of OVX mice

**Figure 2:**
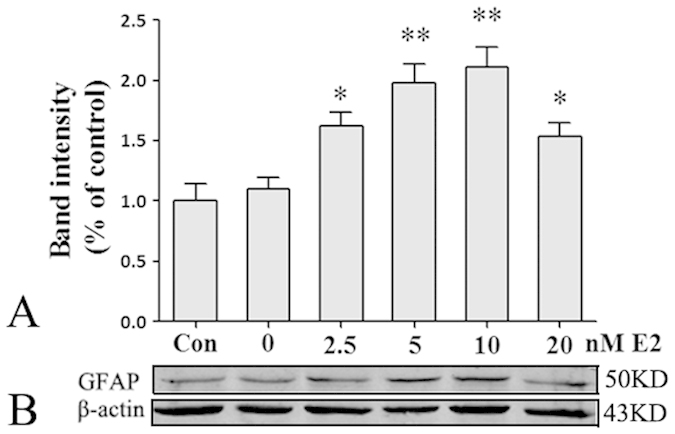
E2 up-regulated GFAP protein expression in cultured astrocytes (*n* = 5). (**A**) Data are shown as the mean ± S.D; *p < 0.05 and **p < 0.01 vs. the 0 nM group. (**B**) Cropped gels and blots showing the effects of different doses of E2 treatment on the GFAP protein expression. The samples derive from the same experiment and that gels/blots were processed in parallel. Full-length blots/gels are presented in Supplementary Fig. 4.

**Figure 3:**
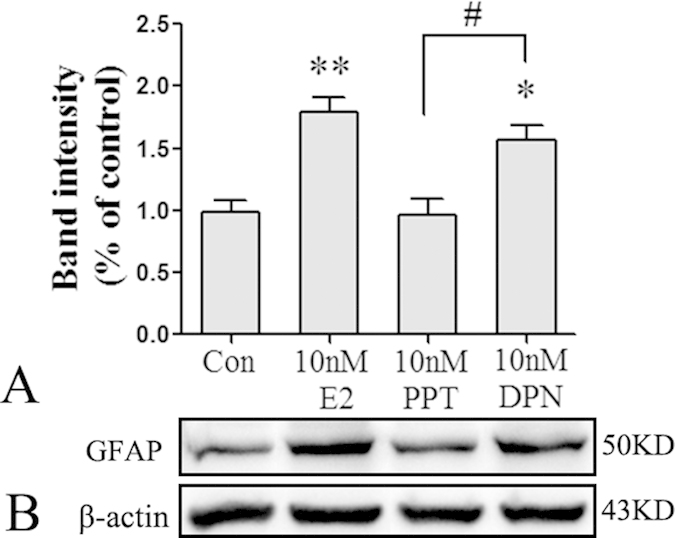
The ERβ agonist DPN increased GFAP protein expression of pure cultured astrocytes (*n* = 5). (**A**) Data are shown as the mean ± S.D; *p < 0.05 and **p < 0.01 vs. Con group; ^#^p < 0.05 vs. PPT group. (**B**) Cropped gels and blots showing the expression of GFAP protein in various group. The samples derive from the same experiment and that gels/blots were processed in parallel. Full-length blots/gels are presented in Supplementary Fig. 4.

In cultured astrocytes, E2, PPT(ERα agonist) and DPN(ERβ agonist) stimulation were used to assess the effects of E2 on GFAP expression. Treatment with E2 significantly increased GFAP protein expression in a concentration-dependent manner with a maximum concentration of 10 nM (Fig. 2; *p < 0.05, **p < 0.01 vs. the 0 nM group). PPT and DPN were applied to cultured astrocytes. As shown in Fig. 3, 10 nM PPT did not significantly increase GFAP protein expression, whereas 10 nM E2 and 10 nM DPN significantly increased GFAP protein expression(*p < 0.05, **p < 0.01 vs. the control (Con) group; ^#^p < 0.05 vs. the 10 nM PPT group).

In the *in vivo* experiments, the OVX model and E2 replacement treatment were used to assess the effects of E2 on GFAP expression in the mouse hippocampus. The OVX status and E2 replacement were further validated by determining the serum E2 levels. As shown in Supplementary Fig. 2B, the levels of serum E2 in the Con group (30.15 ± 3.54 pg/ml) further indicated that the mice were in diestrus. The level of serum E2 in the OVX group (16.6 ± 2.93 pg/ml) was significantly lower than that in the Con group (*p < 0.05). Furthermore, the levels of serum E2 in the mice that received 50 μg/kg E2 replacement (39.27 ± 4.24 pg/ml) successfully raised the relatively low levels of E2 shown by OVX mice (^##^p < 0.01 vs. the OVX group).

**Figure 4:**
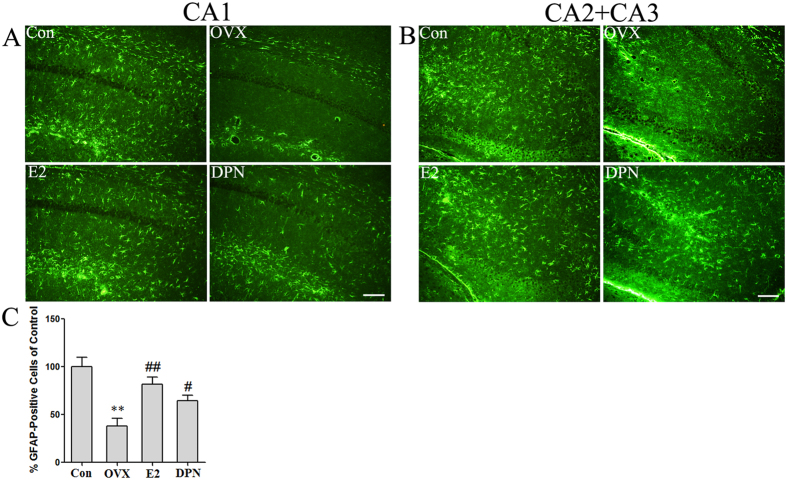
E2 and DPN replacement increased the number of GFAP-positive cells in the hippocampus (*n* = 5). An immunofluorescence assay revealed that in CA1 region (**A**), CA2 and CA3 region (**B**) of hippocampus, the number of GFAP-positive cells in the OVX mice was significantly lower than that in the normal mice; and 50 μg/kg E2 and 8 mg/kg DPN treatment significantly increased the number of GFAP-positive cells. (**C**) Data are shown as the mean ± S.D; **p < 0.01 vs. Con group; ^#^p < 0.05 and ^##^p < 0.01 vs. OVX group. Bar: 50 μm.

Using an immunofluorescence assay, the number of GFAP-positive cells of the OVX mice was significantly decreased to 38.0% ± 8.0% compared with the normal mice (**p < 0.01). In the CA1 region (Fig. 4A) and the CA2 and CA3 regions (Fig. 4B) of the hippocampus, 50 μg/kg E2 and 8 mg/kg DPN replacement, respectively, significantly increased the number of GFAP-positive cells to 81.7% ± 7.6% and 64.3% ± 6.0% (^#^p < 0.05, ^##^p < 0.01 vs. the OVX group). No significant difference was observed between the E2 and DPN groups.

**Figure 5:**
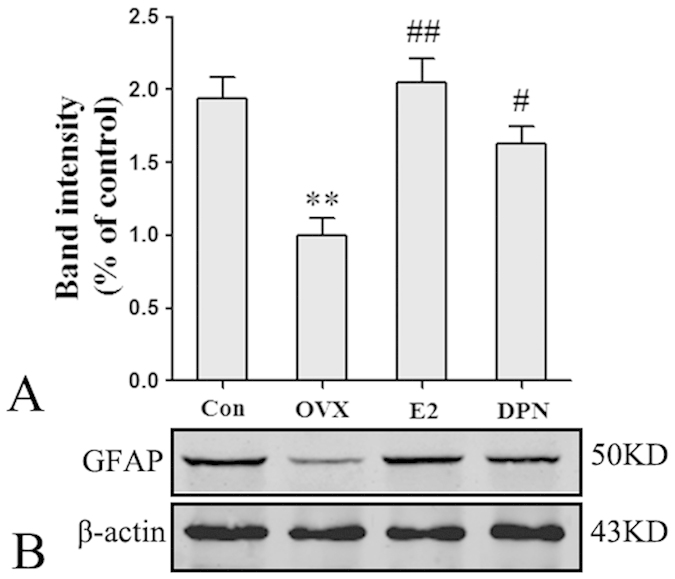
E2 and DPN replacement up-regulated GFAP protein expression in the mice hippocampus (*n* = 5). (**A**) Data are shown as the mean ± S.D; **p < 0.05 vs. Con group; ^#^p < 0.05 and ^##^p < 0.01 vs. OVX group. (**B**) Cropped gels and blots showing the expression of GFAP in 50 μg/kg E2 and 8 mg/kg DPN replacement group. The samples derive from the same experiment and that gels/blots were processed in parallel. Full-length blots/gels are presented in Supplementary Fig. 4.

The results of western blot analyses corresponded to those of the immunofluorescence assay. As shown in Fig. 5, the expression levels of GFAP protein in the OVX group were significantly decreased compared with those of the Con group (**p < 0.01), and 50 μg/kg E2 and 8 mg/kg DPN replacement significantly increased GFAP protein expression (^#^p < 0.05 and ^##^p < 0.01 vs. the OVX group). No significant difference was observed between the E2 and DPN groups.

### Astrocytes mediated the neuroprotection of E2 on neuronal apoptosis induced by OGD and reperfusion

**Figure 6:**
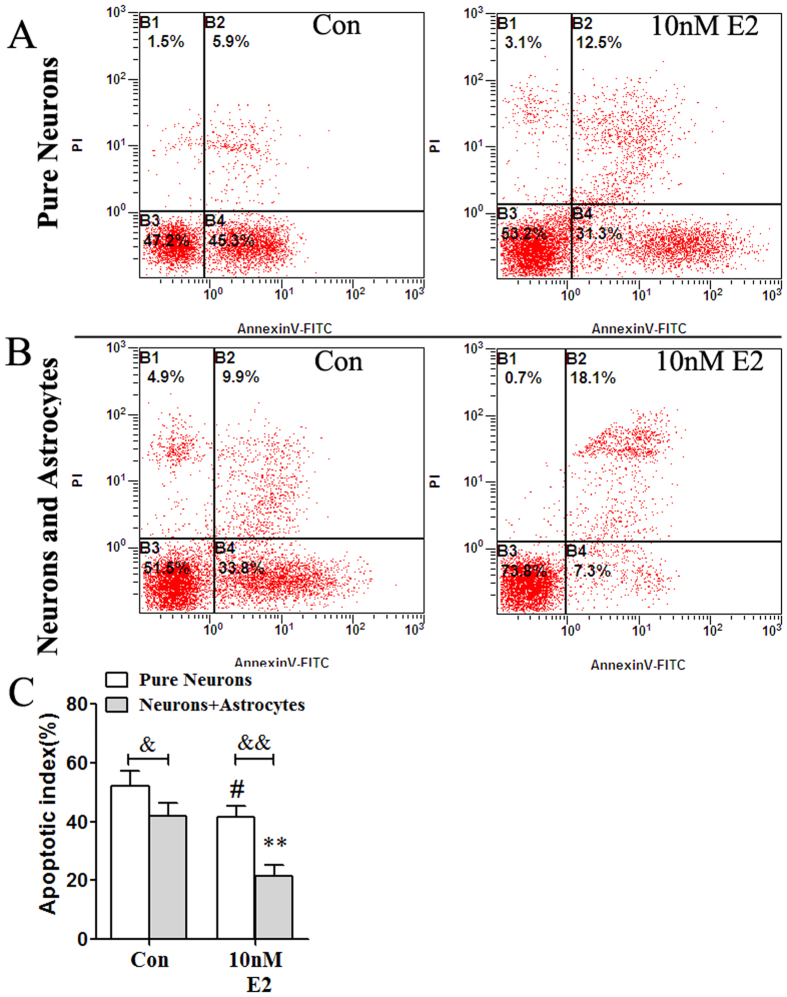
Astrocytes mediated the neuroprotection of E2 on neuronal apoptosis induced by OGD and reperfusion (*n* = 5). (**A,B**) Representative photographs showing neuron apoptosis induced by OGD and reperfusion in pure neurons groups (**A**) or neurons and astrocytes cocultured groups (**B**). (**C**) Data are shown as the mean ± S.D; *p < 0.05 and **p < 0.01 vs. Con group; ^&^p < 0.05, ^&&^p < 0.01.

Flow cytometric analysis was used to evaluate the effect of E2 on neuronal apoptosis following exposure of the pure neurons or cocultured cells to OGD-R (Fig. 6). In pure neurons, the apoptotic index of the Con group was 52.3% ± 4.9%. Pretreatment with 10 nM E2 attenuated OGD-R-induced cell apoptosis to values of 41.6% ± 3.7% (^#^p < 0.05 vs. the Con group). In neurons and astrocytes cocultures, the apoptotic index of the Con group was 41.8% ± 4.4%, with a significant decrease compared with the pure neurons Con group (^&^p < 0.05). Pretreatment with 10 nM E2 attenuated OGD-R-induced cell apoptosis to values of 21.6% ± 3.6% (**p < 0.01 vs. the Con group), with a significant decrease compared with the pure neurons 10 nM E2 group (^&&^p < 0.01).

### E2 attenuated neuronal apoptosis via astrocytes ERβ

**Figure 7:**
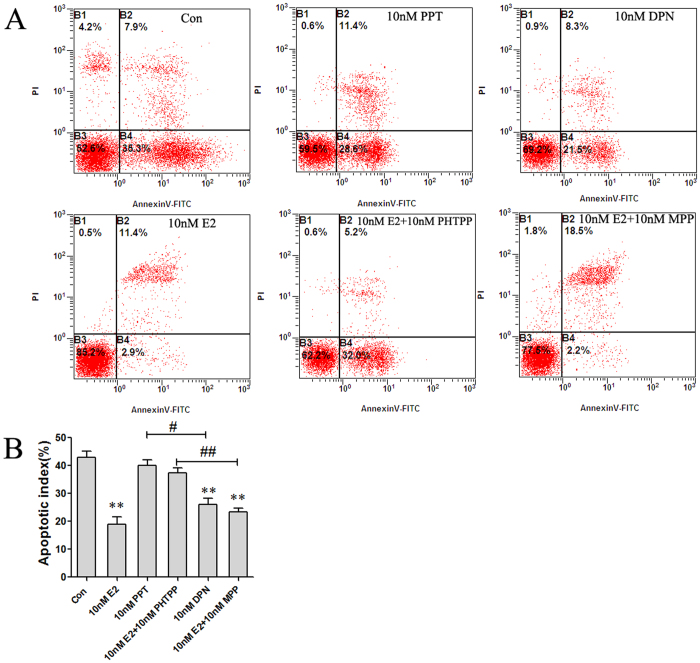
E2 attenuated neuronal apoptosis via astrocytes ERβ (*n* = 5). (**A**) Representative photographs showing neuron apoptosis induced by OGD and reperfusion in cocultured neurons and astrocytes of Con, 10 nM E2, 10 nM PPT, 10 nM E2+10 nM PHTPP, 10 nM DPN or 10 nM E2+10 nM MPP group. (**B**) Data are shown as the mean ± S.D; **p < 0.01 vs. Con group; ^&^ < 0.05, ^&&^ < 0.01; ^#^p < 0.05, ^##^p < 0.01.

As shown in Fig. 7, in neurons and astrocytes cocultures, the apoptotic index of the Con group was 42.9% ± 3.9%. Pretreatment with 10 nM E2 attenuated OGD-R-induced cell apoptosis to values of 18.9% ± 4.6% (**p < 0.01 vs. the Con group). Pretreatment with 10 nM PPT or 10 nM E2+10 nM PHTPP (ERβ antagonists) did not attenuated OGD-R-induced cell apoptosis. Whereas, pretreatment with 10 nM DPN, or 10 nM E2+10 nM MPP (ERα antagonists) attenuated OGD-R-induced cell apoptosis to values of 25.9% ± 4.2% and 23.3% ± 2.5%, respectively (**p < 0.01 vs. the Con group; ^#^p < 0.05 vs. 10 nM PPT; ^##^p < 0.01 vs. 10 nM E2+10 nM PHTPP group). No significant difference was observed between the Con group, the 10 nM DPN group and the 10 nM E2+10 nM MPP group.

**Figure 8:**
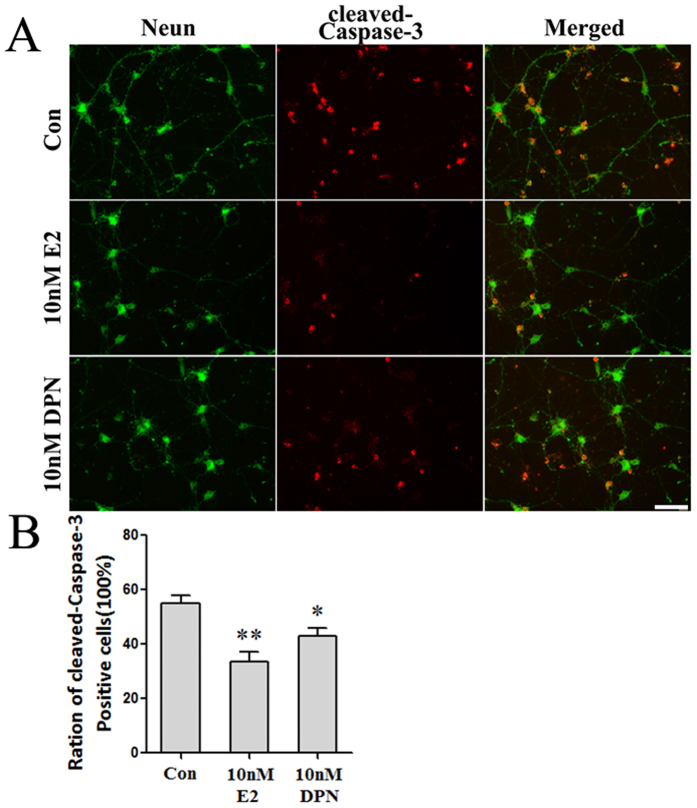
Pre-treatment with E2 and DPN decreased the number of cleaved-Caspase-3-positive neurons after OGD and reperfusion (*n* = 5). (**A**) Representative photographs showing the cleaved-Caspase-3-positive neurons Con, 10 nM E2 or 10 nM DPN group. Bar: 10 μm. (**B**) Data are shown as the mean ± S.D; *p < 0.05 and **p < 0.01 vs. Con group.

**Figure 9:**
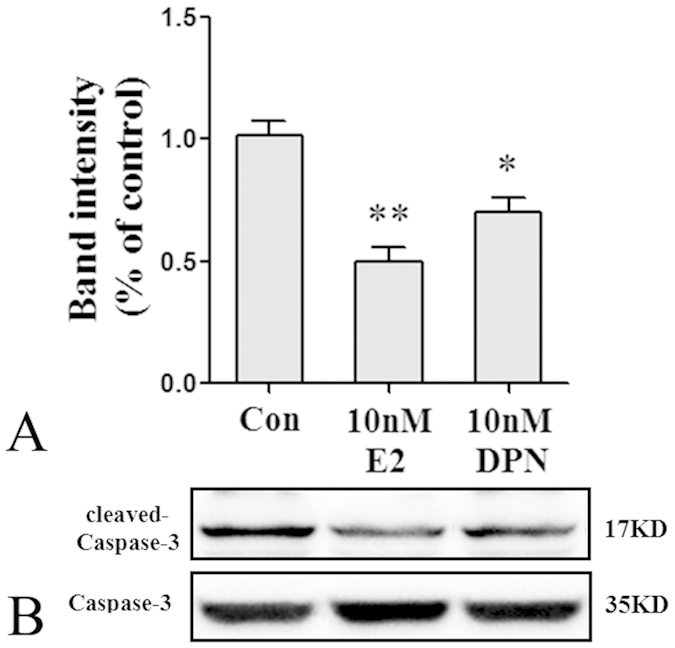
Pre-treatment with E2 and DPN decreased cleaved-Caspase-3 protein expression in neurons received OGD and reperfusion (*n* = 5). (**A**) Data are shown as the mean ± S.D; *p < 0.05 and **p < 0.01 vs. Con group. (**B**) Cropped gels and blots showing the expression of cleaved-Caspase-3 protein in Con, 10 nM E2 or 10 nM DPN group. The samples derive from the same experiment and that gels/blots were processed in parallel. Full-length blots/gels are presented in Supplementary Fig. 4.

Next, we detected the effects of estrogen and DPN on the expression of cleaved-Caspase-3 protein, which is an apoptosis marker. The results analyzed by immunofluorescence and western blot assays corresponded to those of the flow cytometric analysis. As shown in Fig. 8, an immunofluorescence assay indicated that the proportion of cleaved-Caspase-3-positive cells was 55.0% ± 2.9% in the Con group. Pretreatment with 10 nM E2 or 10 nM DPN significantly decreased the proportions of cleaved-Caspase-3-positive cells to 33.7% ± 3.5% and 43.0% ± 2.9%, respectively (^#^p < 0.05, ^##^p < 0.01 vs. the Con group). No significant difference was observed between the 10 nM E2 group and the 10 nM DPN group. Western blot analysis (Fig. 9) confirmed that pretreatment with 10 nM E2 or 10 nM DPN significantly decreased the expression levels of cleaved-Caspase-3 protein compared with that of the Con group (*p < 0.05 and **p < 0.01). No significant difference was observed between the 10 nM E2 group and the 10 nM DPN group.

### E2 and DPN replacement treatment alleviated neuronal apoptosis induced by global cerebral ischemia (GCI) and reperfusion

In the *in vivo* experiments, a GCI model was used to evaluate the neuroprotective effects of E2 and DPN treatment. The physiological parameters of the animals during the GCI period are summarized in Supplementary Table 1. The blood pressure and blood gas values remained within the normal range, and no significant differences in the physiological parameters were observed among the groups. The GCI surgeries were performed with regional cerebral blood flow monitoring. As shown in Supplementary Fig. 3, the cerebral blood flow in the mice was reduced to <10% of the baseline level during the 20-min period of transient occlusion, which is generally considered to represent a successful GCI model.

**Figure 10:**
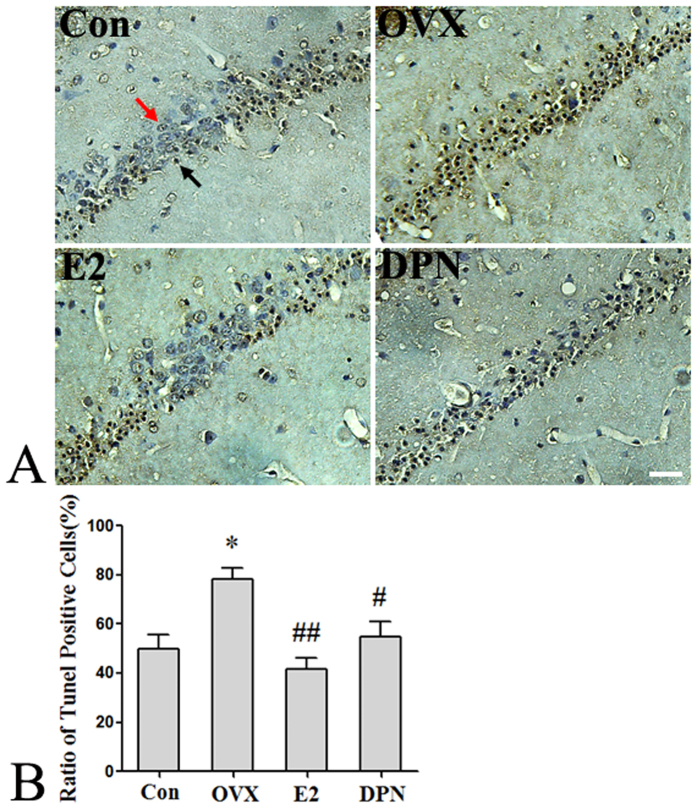
E2 and DPN replacement reduced the number of TUNEL-positive cells in hippocampus CA1 region 24 h after reperfusion (*n* = 5). (**A**) Representative photographs showing neuronal damage in CA1 region. The red arrow represents the living neurons with nuclei stained blue and the black arrow represents TUNEL-positive cells which shows a pyknotic and sepia nuclei. Bar: 50 μm. (**B**) Data are shown as the mean ± S.D; *p < 0.05 vs. Con group; ^#^p < 0.05 and ^##^p < 0.01 vs. OVX group.

Three days after the GCI surgeries, we assessed neuronal apoptosis in the hippocampus. As shown in Fig. 10, the proportion of TUNEL-positive cells in the CA1 region of the hippocampus was 50.5% ± 5.8% in the Con group, and the OVX group exhibited a significant increase in the proportion of TUNEL-positive cells to 78.33% ± 4.41% (*p < 0.05 vs. the Con group). Treatment with 50 μg/kg E2 and 8 mg/kg DPN significantly decreased the proportion of TUNEL-positive cells to 41.7% ± 4.4% and 55.3% ± 5.8%, respectively (^#^p < 0.05, ^##^p < 0.01 vs. the OVX group). No significant difference was observed between the E2 and DPN groups.

**Figure 11:**
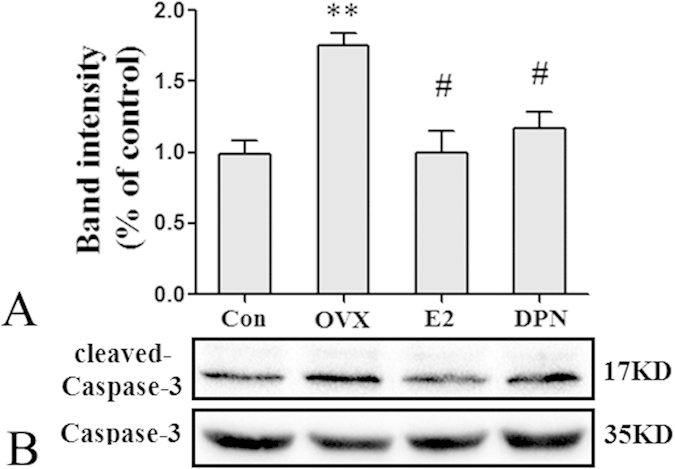
E2 and DPN replacement decreased cleaved-Caspase-3 protein expression in hippocampus 24h after reperfusion (*n* = 5). (**A**) Data are shown as the mean ± S.D; *p < 0.05 vs. Con group; ^#^p < 0.05 vs. OVX group. (**B**) Cropped gels and blots showing the expression of cleaved-Caspase-3 protein in Con, OVX, E2 treatment or DPN treatment group. The samples derive from the same experiment and that gels/blots were processed in parallel. Full-length blots/gels are presented in Supplementary Fig. 4.

The results of western blot analyses of cleaved-Caspase-3 protein expression, corresponded to those of the TUNEL staining. As shown in Fig. 11, the expression levels of cleaved-Caspase-3 protein in the OVX group were significantly increased compared with those of the Con group (**p < 0.01), and 50 μg/kg E2 and 8 mg/kg DPN replacement significantly decreased cleaved-Caspase-3 protein expression (^#^p < 0.05 vs. the OVX group). No significant difference was observed between the E2 and DPN groups.

**Figure 12:**
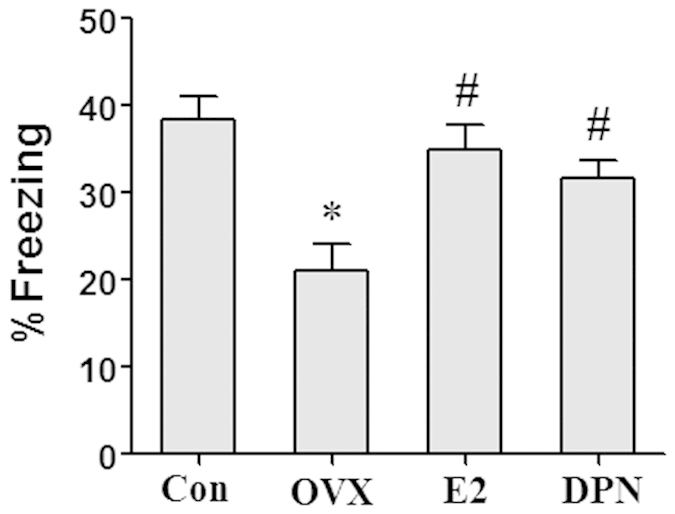
E2 and DPN replacement improved hippocampal-dependent contextual fear response 7 days after reperfusion (*n* = 6). Data are shown as the mean ± S.D; *p < 0.05 vs. Con group; ^#^p < 0.05 vs. OVX group.

Seven days after GCI surgery, we performed fear conditioning test which is a hippocampal-dependent cognitive testing to evaluate hippocampus neuronal injury. As shown in Fig. 12, the OVX group exhibited significantly impaired hippocampal-dependent contextual fear response than the Con group (*p < 0.05). Treatment with 50 μg/kg E2 and 8 mg/kg DPN significantly improved the contextual fear response compared with the OVX group (^#^p < 0.05). No significant difference was observed between the E2 and DPN groups.

**Figure 13:**
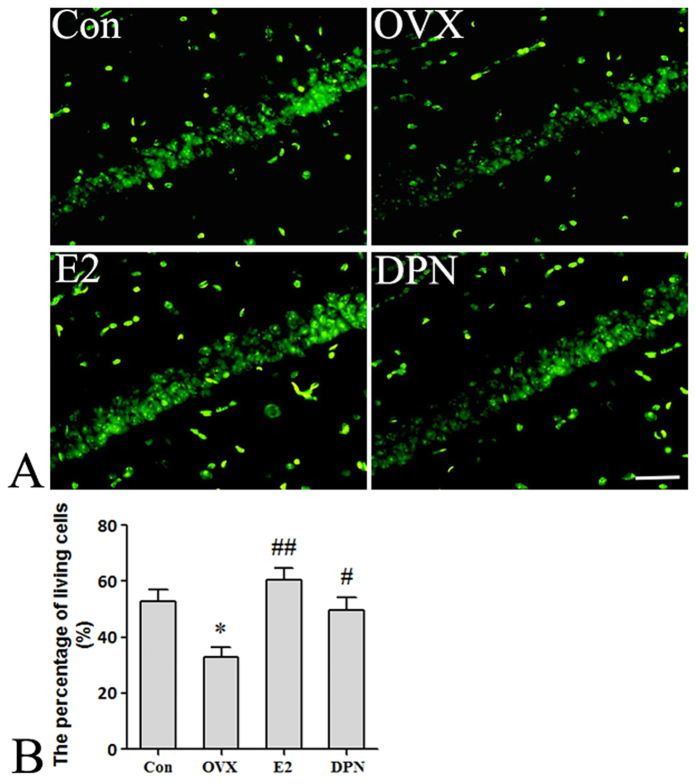
E2 and DPN replacement increased the number of Neun-positive cells in hippocampus CA1 region 7 days after reperfusion (*n* = 5). (**A**) Representative photographs showing Neun-positive cells in CA1 region. (**B**) Data are shown as the mean ± S.D; *p < 0.05 vs. Con group; ^#^p < 0.05 and ^##^p < 0.01 vs. OVX group. Bar: 50 μm.

Then we assessed neuronal survival in the hippocampus using immunofluorescence assays. As shown in Fig. 13, the proportion of Neun-positive cells in the CA1 region of the hippocampus was 52.8% ± 7.5% in the Con group, and the OVX group exhibited a significant decrease in the proportion of Neun-positive cells to 32.8% ± 6.1% (*p < 0.05 vs. the Con group). Treatment with 50 μg/kg E2 and 8 mg/kg DPN significantly increased the proportions of Neun-positive cells to 60.6% ± 6.9% and 49.5% ± 8.3%, respectively (^#^p < 0.05, ^##^p < 0.01 vs. the OVX group). No significant difference was observed between the E2 and DPN groups.

## Discussion

Several studies, including ours, have demonstrated the neuroprotective effects of ERT against ischemic stroke in either OVX rats or OVX mice^2^,^3^,^4^. However, two clinical trials of ERT have ended unsuccessfully^5^,^6^. Therefore, researchers have recently returned to animal studies to re-examine the underlying mechanism of ERT, with the expectation of identifying new targets of estrogen neuroprotection. We found that many previous studies have focused on the molecular mechanisms of neuronal cell death. However, astrocytes and microglial cells also play important roles in estrogen neuroprotection^7^.

Estrogen exerts its function by mainly binding to ERα and ERβ. Our study demonstrated that both ERα and ERβ are expressed in cultured astrocytes derived from the hippocampus, which is consistent with previous studies^11^,^12^,^16^. These results indicate that astrocytes are a major cellular target of estrogen. Using *in vivo* experiments, we found that ERα and ERβ were highly expressed in the hippocampus of adult female mice, which is consistent with a previous study^17^. However, unexpectedly, we found that hippocampal astrocytes primarily expressed ERβ, both *in vivo* and *in vitro*, under basal conditions. Due to the expression levels of ERβ, it is likely that ERβ plays an important role in the maintenance of astrocytic physiological functions in the hippocampus.

GFAP is considered a specific marker of astrocyte activation. Using *in vitro* experiments, we used different physiological concentrations of E2 to stimulate hippocampal primary astrocytes and found that treatment with E2 significantly increased GFAP protein expression in a concentration-dependent manner with a maximum concentration of 10 nM. Previous studies have identified a functional estrogen response element in the 5′-upstream region of the GFAP promoter and have shown that 1 pM E2 treatment increased GFAP transcription in both monotypic astrocytic cultures and mixed glial cultures^18^,^19^. But in direct cocultures of mixed glia (astrocytes: microglia, 3:1) from rats cortex and neurons from E18 rats, 100 pM E2 enhanced neurite outgrowth and repressed GFAP expression^20^,^21^, which indicate that the effect of E2 on neurons may influence astrocytic GFAP expression. In animal experiments, on the afternoon of proestrus, when plasma estradiol levels were the highest, both GFAP tRNA and mRNA of normal cycling rats were increased in the arcuate nucleus of the hypothalamus^19^. However, the effects of E2 on GFAP expression in the hippocampus have not been well documented. Our study found numerous GFAP-positive astrocytes and high GFAP protein expression levels in the hippocampus of normal adult female mice, whereas few GFAP-positive cells and minimal GFAP expression were observed in OVX mice, which had very low serum estrogen levels. These results demonstrate that the hippocampal astrocytes of OVX mice are in an inactive state and that a normal serum estrogen level is essential to maintain astrocyte activation in the hippocampus of female mice. We also found that treatment of OVX mice with 50 μg/kg E2 for 3 weeks not only maintained the serum estrogen level at 39.27 ± 4.24 pg/ml, which was similar to that of the Con group, but also significantly increased the number of GFAP-positive astrocytes and the level of GFAP protein expression compared with the OVX group. According to these results, we conclude that estrogen can upregulate GFAP expression in astrocytes both *in vivo* and *in vitro*.

However, the type of estrogen receptor (ERα or ERβ) that mediates this regulation remains unclear. PPT is the first selective agonist for the ERα subtype to be developed and exhibits a 410-fold binding selectivity over ERβ^22^, whereas DPN acts as an agonist on both ER subtypes but exhibits a 70-fold higher relative binding affinity and 170-fold higher relative estrogenic potency in transcription assays with ERβ than with ERα^23^. In cultured astrocytes, 10 nM DPN, but not 10 nM PPT, significantly increased GFAP protein expression. Using *in vivo* experiments, we treated OVX mice with 8 mg/kg DPN for 3 weeks and found that DPN replacement significantly increased the number of GFAP-positive astrocytes and the level of GFAP protein expression. These results demonstrate for the first time that DPN upregulated astrocyte GFAP expression and that estrogen activated astrocytes via ERβ. N-Myc downstream-regulated gene 2 (NDRG2) has been recognized as a new specific marker for astrocytes^24^ and plays several important roles in astrocyte function. Our previous study found that estrogen upregulated NDRG2 mRNA and protein expression via ERβ both *in vivo* and *in vitro*^25^. According to these results, we propose that ERβ is a key mediator of multiple effects of estrogen on astrocytic physiological function.

We found that pretreatment of astrocyte and neuronal cocultures with 10 nM E2 significantly attenuated OGD-R-induced neuronal apoptosis compared to pure neurons with 10 nM E2, which demonstrated that estrogen-induced neuroprotection against ischemic nerve injury involved the activation of astrocytes. Although physiological concentrations of E2 were not consistently protective in purified neuronal cultures, E2 was protective when neurons were cultured in the presence of astrocytes. Using *in vivo* experiments, our study found that the OVX mice with astrocytes exhibited severe neuronal apoptosis, and E2 replacement markedly alleviated this neuronal injury, demonstrating that E2 replacement exerted significant neuroprotective effects against GCI. Our previous studies have verified that this physiological-concentration E2 replacement significantly reduces brain infarction induced by middle cerebral artery occlusion (MCAO) in both rats and mice^2^,^3^. GCI, arising during cardiac arrest or surgery in humans or induced experimentally in animals, elicits selective, delayed neuronal death; pyramidal neurons of the hippocampal CA1 region are particularly vulnerable^26^. Although several studies have demonstrated the neuroprotective effects of E2 replacement in a GCI model^27^,^28^,^29^, these studies neglected the role of astrocytes in E2 replacement-mediated neuroprotection.

Proper astrocyte function is particularly important for neuronal survival under ischemic conditions, as astrocytes are involved in several activities that profoundly influence tissue viability during ischemia, including glutamate homeostasis, water balance, maintenance of the blood-brain barrier, cerebral blood flow regulation, ion homeostasis, and secretion of neuroprotective factors^8^. An increase in GFAP in astrocytes indicates the activation of astrocytes. A recent study found that ischemic preconditioning-induced neuroprotection involved the activation of astrocytes^15^. Activated astrocytes enhance the ability of neurons to eliminate excitatory neurotransmitters and ions such as glutamate, H^+^ and K^+^; they also increase the storage of glycogen and the synthesis of cytokines and neurotrophic factors. Therefore, the activation of astrocytes is closely related to ischemic tolerance. In rats subjected to transient forebrain ischemia, CA1 astrocytes lose glutamate transport activity and immunoreactivity for GFAP and glutamate transporter GLT-1 and exhibit increases in mitochondrial free radicals and reduced mitochondrial membrane potential within a few hours of reperfusion, suggesting that the dysfunction of hippocampal CA1 astrocytes is central to the well-documented delayed death of CA1 neurons^26^. Localized photothrombotic/ischemic cortical injury initiates a significant increase in astrocyte production from the subventricular zone, and it is these astrocytes, and not the neuroblasts, that locate the injured cortex and exert neuroprotective effects^30^. We found that E2 replacement significantly reduced the number of TUNEL-positive neurons and the level of cleaved-Caspase-3 expression in the CA1 region. ERT increased Bcl-2 expression in CA1 neurons^27^,^29^, which may have resulted from the activation of astrocytes. According to these results and the role of astrocytes in ischemia, we conclude that the neuroprotective effects of E2 replacement against global ischemia must be partially mediated by astrocytes.

In *in vitro* experiments, by using selective estrogen receptor agonist (PPT and DPN) and antagonists (MPP and PHTPP), we found DPN treatment, not PPT, significantly attenuated OGD-R-induced neuronal apoptosis. In *in vivo* experiments, we found that DPN treatment exhibited significant neuroprotective effects against GCI. Taken together, these results demonstrated that the astrocytes–induced neuroprotective effects of ERT was mediated by ERβ. However, the definitive roles of ERα and ERβ in neuroprotection remain unresolved. ERα mRNA was upregulated in the penumbra region following MCAO in rats, suggesting a possible role for ERα in neuroprotection^31^, although the cell type in which this increase occurs was not determined. Using ERα and ERβ KO mice, Dubal *et al.*^32^ found that ERα was required for the protective effects of estradiol against brain injury. Additionally, in EAE, estrogen mediates neuroprotection and anti-inflammatory effects through ERα in astrocytes but not through ERβ in astrocytes or neurons^14^,^33^. And Guo *et al.* found that E2 provides direct protection to astrocytes from OGD-R injury by an ERα not ERβ-dependent mechanism^34^. However, it has been demonstrated that ERβ-KO mice experience a significant loss of neurons coupled with astroglia proliferation in the cerebral cortex^35^, suggesting a role for ERβ in basal neuronal maintenance. Carswell *et al.*^36^ found that pretreatment with DPN, but not PPT, significantly reduced ischemic neuronal damage. In hippocampal slice cultures, pretreatment with E2 (10 nM) for 7 days caused a 25% increase in ERβ protein expression and a 20% reduction in ERα protein expression and significantly protected the CA1 area against OGD, suggesting that estrogen-induced neuroprotection against ischemia involves the regulation of ERβ^37^. Long-term periodic ER-β agonist treatment improves post-ischemic outcome and cognition in the hippocampus of OVX female rats, which involves the upregulation of p-CREB expression^38^. Given the reported widespread expression of ERβ, but not ERα, in the normal adult hippocampus, ERβ likely mediates at least some of the physiological actions of E2 in the hippocampus. ERβ agonist replacement increased key synaptic proteins *in vivo*, including PSD-95, synaptophysin and the AMPA-receptor subunit GluR1, which demonstrates that the effects of estrogen on hippocampal synaptic plasticity and memory are mediated by ERβ^39^. Nevertheless, other studies have indicated that both ERα and ERβ contribute to estrogen-induced neuroprotection^28^,^40^. These results suggest that ERα may be more important in injury-induced E2-mediated protection, whereas ERβ may play an important role in basal neuroprotection.

Additionally, several researches have demonstrated that estrogen exhibit neuroprotection by inhibiting microglia-induced inflammation^41^,^42^, which has not been explored in this work. However, the role and expression of ERα and ERβ in microglia are particularly controversial. Some studies have found that microglial cells express ERβ, but not ERα, and that ERβ mediates anti-inflammatory effects^43^,^44^,^45^. Other studies have observed ERα in microglia, which may also have anti-inflammatory neuroprotective effects^46^.

We conclude that ERT-induced neuroprotection against global ischemia involves the activation of astrocytes via ERβ. The clinical trial on ERT was interrupted prematurely due to findings of increased risks of coronary heart disease and breast cancers^5^. As ERβ is highly expressed in the brain and exhibits little or no expression in the breast or uterus^40^, the identification and design of selective ERβ modulators would provide a new strategy to promote the beneficial effects of estrogen in the brain without activating the undesirable effects of estrogen in the reproductive organs, thus avoiding the peripheral risks associated with ERT. However, the effects and mechanisms of selective ERβ modulators in neuroprotection require further validation and exploration.

## Materials and Methods

### Primary astrocyte culture

Briefly, meninges-free brain hippocampus tissue was collected from 1-day-old C57BL/6 mice. Cells were dispersed using mechanical and enzymatic dissociation and a solution containing 0.025% trypsin (Invitrogen, USA). The cells were then suspended in plating medium, including Dulbecco’s modified Eagle’s medium (DMEM; Gibco, USA) containing 10% fetal bovine serum (FBS, Gibco) and 0.5% penicillin/50 U streptomycin. The cells were seeded onto 75 cm^2^ flasks, which were coated with 25 μg/ml poly-L-lysine (Sigma-Aldrich, USA) 1 day prior. The cultures were incubated at 37 °C in a 95%/5% mixture of atmospheric air and CO_2_, respectively. The culture medium was changed once every 3 days. After 7 days, the flasks were put onto a thermostatic shaker at 37 °C and 200 rpm/min for 18 h. Then the cells were seeded onto 6-well plates with or without glass coverslips for drug treatment or immunofluorescence assays.

To identify astrocytes, the presence of GFAP (a specific marker for astrocytes) was assayed in the cultured cells using mouse anti-GFAP antibody^47^ (GA5, 1:1000, Cell Signaling Technology, USA) and DAPI (Roche, Switzerland). This analysis revealed that at least 95% of cells in the culture were GFAP-positive (Supplementary Fig. 1).

### Indirect primary neuron–astrocyte co-culture

Embryonic brains isolated from pregnant 13–14d C57 mice were used to culture primary hippocampal neurons. The procedure is as the same as primary astrocyte cultures, except that Neurobasal media (Gibco) with 1% Glutamate and 2% B27 were used to culture primary neurons. The neurons were cultured in 6-well plates with or without glass coverslips. At the first day of culture, 5 μM Ara-C was added into neuron cultures to reduce glial cell contamination. Then transwells (0.4 μm, Coring, USA) with astrocyte cultures were placed on top of matured neuron cultures. The co-cultures were incubated at 37 °C in a 95%/5% mixture of atmospheric air and CO_2_, respectively.

### Drug treatment

Primary astrocytes were treated with 17b-estradiol (E2, Caymen,USA) at physiological concentrations (0 nM, 2.5 nM, 5 nM, 10 nM and 20 nM) as well as 10 nM ERα agonist PPT (4,40,400–[4-propyl-(1H)-pyrazole-1,3,5-triyl] tris-phenol; Tocris Cookson, USA) and 10 nM ERβ agonist DPN (2,3-bis[4-hydroxyphenyl]-propionitrile; Tocris Cookson) for 24 h. Dimethyl sulfoxide (DMSO) alone was used as the vehicle control. Pure neurons and cocultures were pre-treated with 10 nM E2 for 24 h before receiving OGD-R. In addition, the cocultures of neurons and astrocytes were pre-treated with 10 nM E2, 10 nM PPT, 10 nM E2+10 nM ERβ antagonists PHTPP {4-[2-phenyl-5,7-bis (trifluoromethyl) pyrazolo[1,5-a] pyri midin-3-yl] phenol, Tocris Cookson} and 10 nM DPN or 10 nM E2+10 nM ERα antagonists MPP {1,3-Bis(4-hydroxyphenyl)-4-mtehyl-5-[4-(2-piperidiylethoxy)phenol]-1H-pyrazole dihydrochloride methyl-piperidino-pyrazole, Tocris Cookson} for 24 h before receiving OGD-R. All drugs were dissolved in DMSO as 10 mM stock solutions. Further dilutions were made using culture medium. The final concentration of DMSO in the culture medium never exceeded 0.02%, a level that had no effect by itself.

### Oxygen and glucose deprivation and reperfusion (OGD-R)

After 24 h incubation with E2 and DPN, the cells were washed twice in d-Hanks buffer and switched to DMEM without glucose (Gibco) (OGD medium). Then the cells were switched to a modular incubator chamber. The chamber was flushed with 3 L/min of a 95% N2/5% CO2 gas mixture for 15 min at room temperature. The chamber was then sealed and placed in a 37 °C container. OGD was carried out for 2 h. Following the OGD, the cells were incubated with DMEM with glucose for an additional 20 h reperfusion under normal conditions.

### Flow cytometric analysis

After OGD and reperfusion, the apoptosis of neurons was assayed by flow cytometry. Briefly, the cells were washed with 1 × annexin V-FITC binding buffer prior to staining with annexin V-FITC and PI for 15 min at room temperature in the dark. The stained cells were immediately analyzed using flow cytometry. Apoptotic and necrotic cells were quantitated by annexin V binding and PI uptake. The annexin V-FITC+/PI− and annexin V-FITC+/PI+cell populations were considered to respectively represent early and late apoptotic cells. And the apoptosis index was calculated as the sum of early and late apoptotic cell populations.

### Animals

Eighty 6-month-old female C57BL/6 mice (23–25g) were obtained from the Laboratory Animal Center of the Fourth Military Medical University. These mice were randomly divided into 4 groups: (A) control group (Con), (B) OVX without 17β-estradiol (E2) (Cayman, USA) replacement group (OVX), (C) OVX and 50 μg/kg/day E2 replacement group (E2), (D) OVX and 8 mg/kg/day DPN replacement group (DPN). The OVX mice received daily subcutaneous injection of E2 (26.25 μg total dose) and DPN (4.2 mg total dose) (diluted in sesame oil solution) for 3 weeks. The concentrations of E2 and DPN selected for this study are based on effective concentrations administered in previous studies^48^,^49^. The animals were maintained under a 12:12-h light-dark cycle and a temperature of 25 °C. All animal experimental procedures followed a protocol approved by the Ethics Committee for Animal Experimentation and proceeded according to the Guidelines for Animal Experimentation of the Fourth Military Medical University.

### OVX and E2 replacement

OVX was induced by dorsolateral incisions, as previously described^50^. The animals in the sham group were subjected to the same operation, but their ovaries remained intact. Ten days following the operation, as shown in Supplementary Fig. 2A, vaginal smears were taken for 5 days before E2 and DPN replacement treatment to confirm removal of the ovaries and cessation of the estrous cycle^51^. All mice presented diestrus vaginal smears prior to E2 treatment, indicating the successful removal of the ovaries and cessation of the estrous cycle. These animals then received daily subcutaneous injection of E2 and DPN (diluted in sesame oil solution) for 3 weeks. Prior to extraction of the brain tissue, the mice in the Con group were also confirmed to be in diestrus.

### Determination of the serum E2 levels

The levels of serum E2 were measured to confirm the effect of E2 replacement. Briefly, the animals were anesthetized with an overdose of pentobarbital sodium, and blood was collected from the ophthalmic artery. Serum estradiol levels were measured using an estrodiol EIA (ES180S, CalBiotech, CA).

### Global Cerebral Ischemia (GCI) and Regional Cerebral Blood Flow Monitoring

In this study, Bilateral common carotid artery occlusion (BCCAO) was used as a model of global cerebral ischemia^52^. Mice were anesthetized with 3% isoflurane. After induction, the anesthetic was maintained at 1.5% isoflurane, which was delivered via a face mask that was specially devised to fit the front part of the animal’s head. A midline incision was made in the region between the neck and sternum to expose the trachea. Both the right and left common carotid arteries were located lateral to the sternocleidomastoid and both common carotid arteries were carefully separated from the surrounding tissues and vagus nerve. Cerebral ischemia was induced by clamping both the arteries with 2 miniature artery clips. The laser Doppler flow meter (PeriFlux System 5000; Perimed, Stockholm, Sweden) was used to measure regional cerebral blood flow (rCBF) (2–3 mm lateral to the bregma) from the time of anesthetic induction to 5 minutes after reperfusion. Only mice whose mean cortical CBF was reduced to <10% of the preischemic value were used for data analysis. After 20 minutes of cerebral ischemia, the clips were removed from both arteries to allow the reflow of blood through the carotid arteries. The incision was sutured using 4-0 Mersilk. During the surgical procedure, the pericranial temperature was monitored using a temperature probe and maintained at 37.0 °C to 37.5 °C with a heating pad. After surgery, animals were placed in a warm surrounding (30 °C to 33 °C) to avoid biased results from hypothermia. Physiological parameters including rectal temperature, blood pressure, blood gas, and glucose were monitored in each mouse at three time points (before/during and post-preconditioning), that each animal had a femoral artery catheter placed, that sampling volume was 0.2 mL, and that a comparable volume of saline to replace withdrawn blood volume was injected so as to control for the effects of hypovolemia on blood pressure. Samples were then measured using a Bayer Rapidlab 1260 system (Bayer, Leverkusen, Germany).

### TUNEL staining

For detection of *in situ* DNA fragmentation, terminal deoxynucleotidyl transferase-mediated dUTP-biotin nick end labeling (TUNEL) staining was performed using an *In Situ* Cell Death Detection Kit (Roche Diagnostics, Mannheim, Germany) according to the manufacturer’s instructions. TUNEL staining was performed on 5-μm-thick paraffin coronal sections. The sections were treated with 0.3% (v/v) H_2_O_2_ for 20 minutes and then incubated in a TUNEL reaction mixture for 1 hour at 37 °C. The sections were then incubated in converter-peroxidase for 30 minutes at 37 °C. After 3 washes in PBS, sections were developed with 3,3′-diaminobenzidine for 5 minutes at room temperature. The total number of TUNEL-positive neurons in the CA1 region were counted in 3 different fields for each section in a blind manner by light microscopy at × 400 magnification (BX51; Olympus, Tokyo, Japan).

### Western blot

Expression of ERα, ERβ, GFAP, cleaved-Caspase-3 and Caspase-3 protein in primary cultured astrocytes, neurons or hippocampus were measured by western blot. In brief, soluble lysates of samples were mixed with sample buffer and NuPAGE reducing agent (Invitrogen). The extracted proteins were separated using 10% SDS-PAGE and then electrically transferred to polyvinylidene difluoride membranes. Subsequently, the membranes were blocked in 5% nonfat dry milk diluted in TBST for 1 h at room temperature. The western blots were probed with rabbit anti-ERα antibody^53^ (ab75635, 1:500, Abcam), rabbit anti-ERβ antibody^54^ (ab3576, 1:500, Abcam), mouse anti-GFAP antibody (GA5, 1:1000, Cell Signaling Technology), rabbit anti-cleaved-Caspase-3^55^ (#9661, 1:1000, Cell Signaling Technology) antibody, rabbit anti-Caspase-3 antibody^56^ (#9662, 1:1000, Cell Signaling Technology) and mouse β-actin antibody^57^ (#3700, 1:1000, Cell Signaling Technology) overnight at 4 °C. The membranes were then incubated with an IRDye secondary anti-rabbit and anti-mouse antibody (Thermo Scientific, USA) for 2 h. Protein bands were visualized using the LI-COR Odyssey System (LI-COR Biotechnology, USA).

### Immunofluorescence assay

Immunofluorescence studies were performed on primary cultured astrocytes and neurons plated on coverslips or on frozen coronal sections of mice brains before or after ischemia. The primary cultured astrocytes or neurons were plated at a density of 1.5 × 10^4^ cells/well on glass coverslips onto 6-well multiwells. The cells were fixed with 4% paraformaldehyde for 1 h, followed by permeabilization and blocking. The mice brains were fixed via transcardial perfusion with 0.9% cold heparinized saline and 4% paraformaldehyde. Post-fixation, the brains were removed and cryoprotected in 20% sucrose and 30% sucrose solutions. Ten μm thick sections were prepared using a Leica CM1900 frozen slicer. The cell coverslips and hippocampal sections were incubated in 1% H_2_O_2_ for 15 min and 0.3% Triton X-100 for 15 min, with 3×washes in PBS post-incubation between each treatment; then blocked in 5% normal goat serum (NGS; 1 h at RT) and incubated overnight at 4 °C in a humid atmosphere with primary antibodies in the following combination: rabbit anti-ERα antibody (ab75635, 1:100, Abcam), rabbit anti-ERβ antibody (ab3576, 1:100, Abcam), mouse anti-GFAP (GA5, 1:1000, Cell Signaling Technology), rabbit anti-cleaved-Caspase-3 (#9661, 1:1000; Cell Signaling Technology) and mouse anti-Neun (MAB377, 1:1000; Millipore, USA) diluted in 1% NGS. Then, the sections were incubated with mixtures of Alexa 488 (red, Invitrogen) and Alexa-647 (green, Invitrogen)-conjugated donkey anti-rabbit and donkey anti-mouse secondary antibodies for 2 h in the dark at room temperature. Finally, the sections were mounted on slides, viewed and photographed using an Olympus BX51 (Japan) fluorescence microscope.

### Fear conditioning

Conditioned fear was carried out using training and context protocols over the course of two days. On both days mice were initially transferred in their home cages to a control chamber for 30 minutes to acclimate. The mice were then transferred individually in a clean cage to the testing chamber. The mice were first left undisturbed for 2 min. Then they were presented with 5 unsignaled footshocks (1 s duration, 0.5 mA, randomly presented during 3 min period). Then the mice were placed 5 min in the control chamber. Mice were returned to their home cage. Long-term contextual fear memory was evaluated 24 h after conditioning. Mice were placed in the conditioning chamber and exposed to the context for 3 min, and then to the control chamber. Freezing time was measured during every minute of exposure to the context. Freezing was defined as lack of any visible movement except respiration, and it was monitored by visual inspection of the video images.

### Statistical Analyses

The statistical analyses were conducted using SPSS 11.0 for Windows software (SPSS Inc., Chicago, IL). All values, except for total motor scores, are presented as the means ± SD and were analyzed using a one-way analysis of variance (ANOVA). Differences between groups were tested using the Tukey post-hoc test. The total motor scores are expressed as the medians and were analyzed using the Kruskal-Wallis test followed by the Mann-Whitney U test with Bonferroni corrections. Values of p < 0.05 were considered statistically significant.

## Additional Information

**How to cite this article**: Ma, Y. *et al.* Estrogen replacement therapy-induced neuroprotection against brain ischemia-reperfusion injury involves the activation of astrocytes via estrogen receptor β. *Sci. Rep.* **6**, 21467; doi: 10.1038/srep21467 (2016).

## Supplementary Material

Supplementary Information
