# Supplementary material for: Estrogen replacement therapy-induced neuroprotection against brain ischemia-reperfusion injury involves the activation of astrocytes via estrogen receptor β
Source: Sci Rep. 2016 Feb 19;6:21467. doi: 10.1038/srep21467 (PMC4759820; doi:10.1038/srep21467)
Supplement: Supplementary Information [file srep21467-s1.pdf]

Estrogen replacement therapy-induced neuroprotection against brain ischemia-reperfusion injury involves the activation of astrocytes via estrogen receptor β

Yulong Ma[1,*], Hang Guo[1,2*], Lixia Zhang[3,*], Liang Tao[1], Anqi Yin[1], Zhaoyu Liu[1], Yan Li[1], Hailong Dong[1], Lize Xiong[1,†], Wugang Hou[1,†]

[1]Department of Anesthesiology, Xijing hospital, The Fourth Military Medical University, Xi'an 710032, China

[2]Department of Anesthesiology, General Hospital of Chinese PLA Beijing Command, Beijing 100700, China

[3]First Affiliated Hospital to Chinese PLA General Hospital, Beijing 100048, China

* These authors contributed equally to this study.

† To whom correspondence should be addressed.

Email: gangwuhou@163.com and mzkxlz@126.com

Tel.: +86 029 84775343

Fax: +86 029 84775337

Supplementary Figure 1

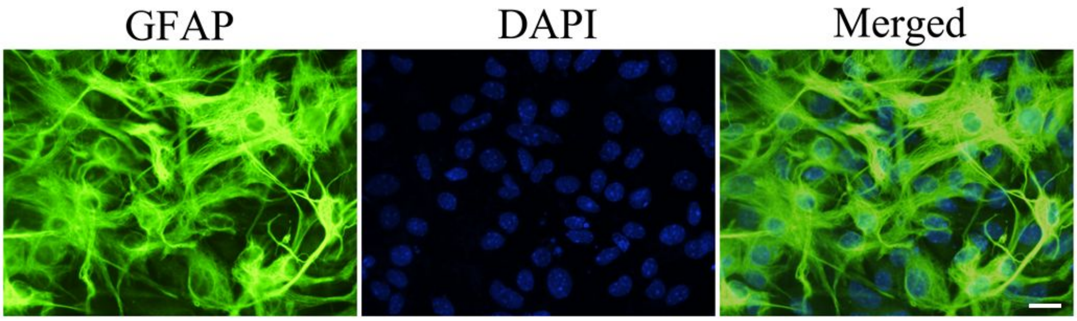

Supplementary figure 1. Identification of primary cultured astrocytes.

GFAP is a specific marker for astrocytes. The analysis revealed that at least 95% of the cultured cells were GFAP-positive. Bar:20 μm.

Supplementary Figure 2

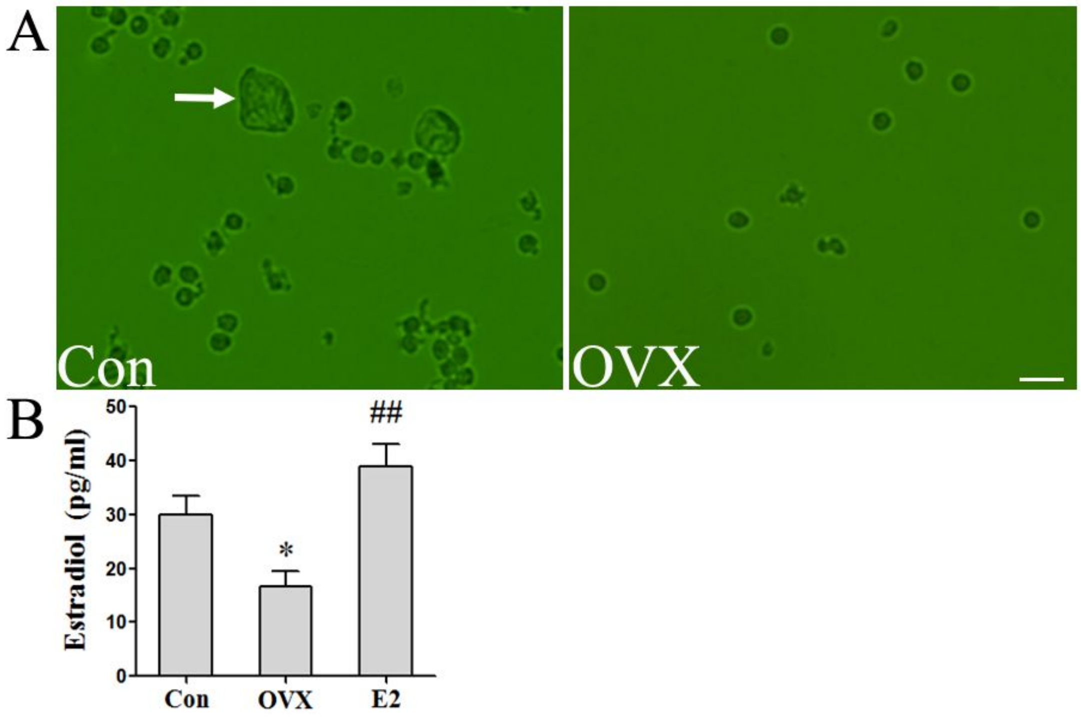

Supplementary figure 2. Verification of OVX status and E2 replacement treatment.

A. Verification of OVX status. The effects of ovary removal and the estrous stage were determined using cytological evaluations of vaginal smears under microscopic examination. The smears of mice in Con group consisted almost exclusively of leukocytes but including several cornified squamous epithelial cells (showed by the white arrow), indicating that these mice were in diestrus. The smears of the OVX mice also consisted of leukocytes, but the number of leukocytes was significantly less than that in Con group, indicating that these mice were also in diestrus. Bar: 20 μm. **B.** Results of the levels of serum E2. Data are shown as the mean±S.D; *p<0.05 vs. Con group; #p<0.05 vs. OVX group.

Supplementary Figure 3

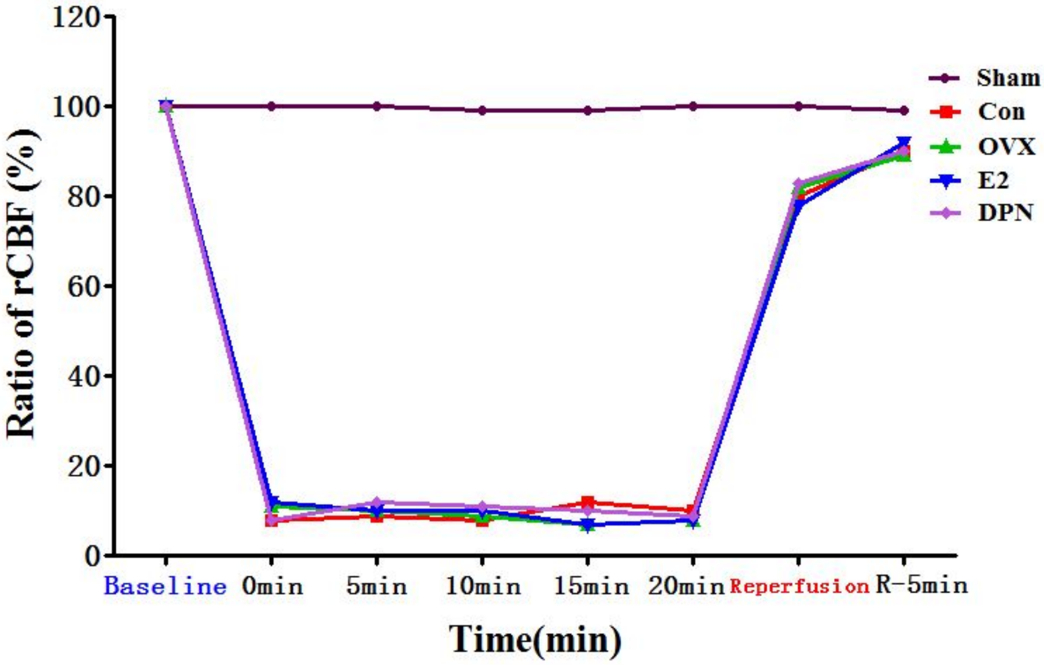

Supplementary figure 3. rCBF Measurements.

The rCBF was immediately reduced to<10 % of the pre-ischemic baseline after BCCAO and remained constant during the ischemic period in all animals. After the clips were removed, and the rCBF returned to pre-ischemic values within 5 min. There were no significant differences in the rCBF between the various groups at any time points.

Supplementary Figure 4

Figure 1D(a)

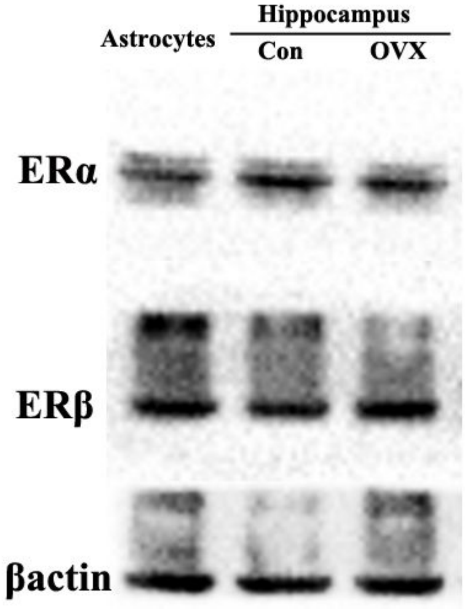

Figure 2B

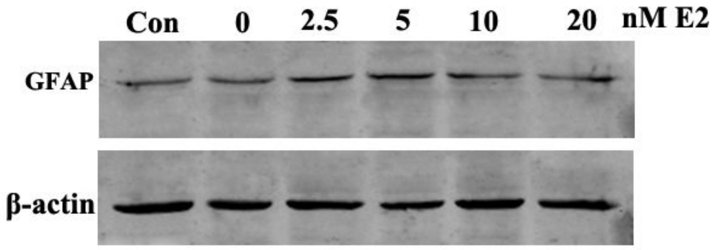

Figure 3B

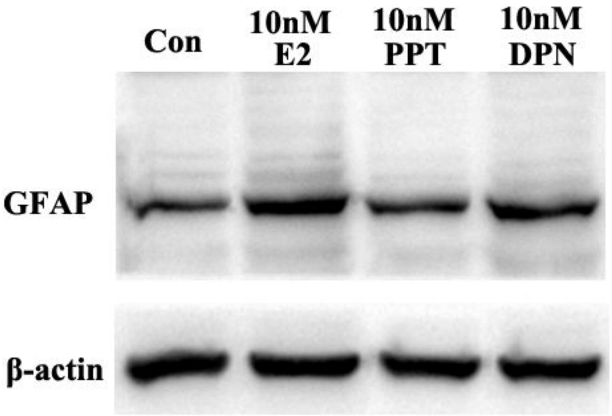

Figure 5B

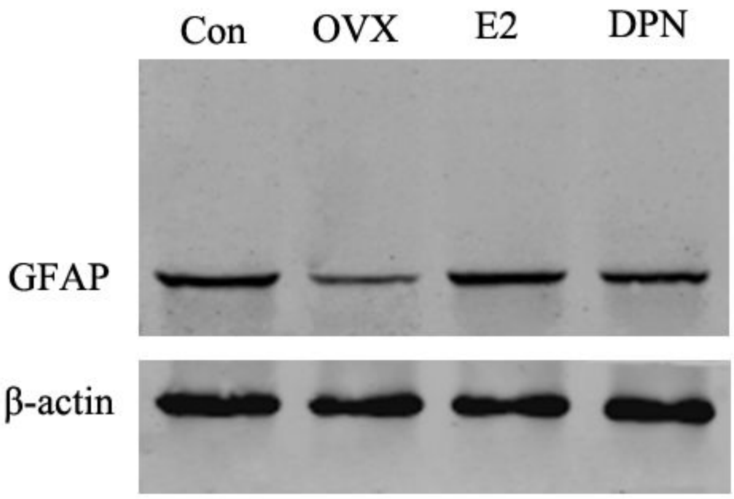

Figure 9B

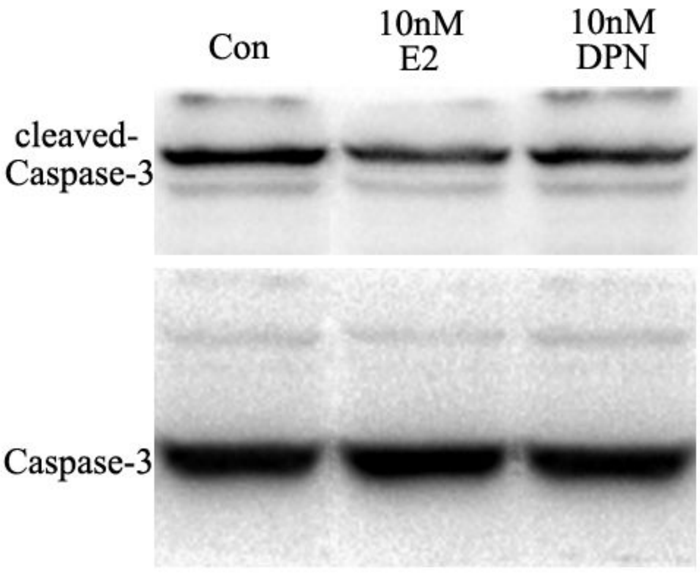

Figure 11B

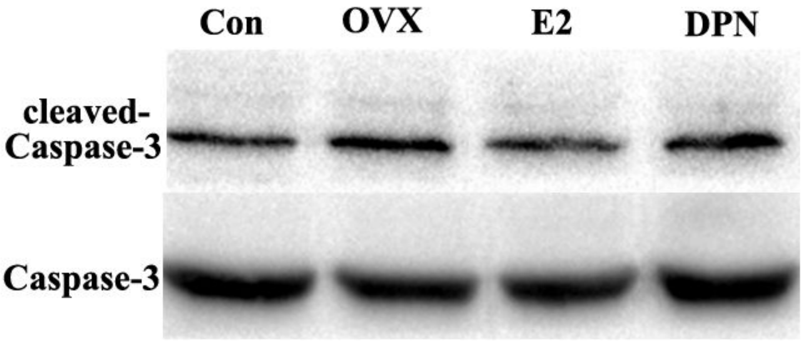

Supplementary figure 4. Full-length blots/gels.

Supplementary table 1

Group		MABP (mmHg)	Temp (ºC)	Glucose (mmol/L)	PH	pO_2 (mmHg)	pCO2 (mmHg)
Con	pre	102±3.8	36.7±0.3	5.76±0.21	7.41±0.03	136.7±3.5	36.1±1.8
	during	104±4.1	36.2±0.4	6.02±0.41	7.39±0.01	133.4±5.2	35.9±2.2
	post	100±3.5	36.5±0.3	5.53±0.33	7.38±0.02	132.6±4.5	36.0±3.0
OVX	pre	103±3.5	37.1±0.2	5.76±0.21	7.41±0.04	132.6±3.8	36.2±2.2
	during	105±5.1	36.4±0.4	6.02±0.41	7.36±0.03	137.5±5.7	35.5±2.4
	post	101±4.5	36.6±0.2	5.53±0.33	7.37±0.05	138.5±4.9	35.7±2.6
50 ug/kg E2	pre	103±4.8	36.9±0.2	5.76±0.21	7.42±0.02	136.5±5.4	36.4±2.7
	during	105±5.1	36.5±0.3	6.02±0.41	7.37±0.02	133.4±4.6	36.0±3.2
	post	102±4.5	36.6±0.4	5.53±0.33	7.39±0.03	135.7±5.4	36.2±2.3
8 mg/kg DPN	pre	103±5.8	36.8±0.4	5.76±0.21	7.40±0.01	136.7±4.3	36.4±1.8
	during	105±2.1	36.5±0.3	6.02±0.41	7.37±0.2	135.4±5.6	36.7±2.0
	post	103±4.5	36.6±0.2	5.53±0.33	7.35±0.3	136.5±5.3	36.5±2.3

MABP: mean arterial blood pressure; Temp: rectal temperature.

Supplementary table 1. The physiological parameters in animals of different groups before, during and after the GCI.

The physiological parameters of OVX, 50 μg/kg E2 and 8 mg/kg DPN groups had no significant difference compared to Con group ($p > 0.05$).
